# Cluster-based segmentation of dual-echo ultra-short echo time images for PET/MR bone localization

**DOI:** 10.1186/2197-7364-1-7

**Published:** 2014-06-04

**Authors:** Gaspar Delso, Konstantinos Zeimpekis, Michael Carl, Florian Wiesinger, Martin Hüllner, Patrick Veit-Haibach

**Affiliations:** GE Healthcare, Waukesha, WI 53186 USA; Department Medical Radiology, Institute of Diagnostic and Interventional Radiology, University Hospital Zurich, Zurich, 8091 Switzerland; Department Medical Radiology, Division of Nuclear Medicine, University Hospital Zurich, Rämistrasse 100, Zurich, 8091 Switzerland; GE Global Research, Munich, 85748 Germany

**Keywords:** PET/MR, Attenuation correction, Bone, UTE, Dual-echo

## Abstract

**Background:**

Magnetic resonance (MR)-based attenuation correction is a critical component of integrated positron emission tomography (PET)/MR scanners. It is generally achieved by segmenting MR images into tissue classes with known attenuation properties (e.g., bone, fat, soft tissue, lung, air). Ultra-short echo time (UTE) have been proposed in the past to locate bone tissue. In this study, tri-modality computed tomography data was used to develop an improved algorithm for the localization of bone in the head and neck.

**Methods:**

Twenty patients were scanned using a tri-modality setup. A UTE acquisition with 22-cm transaxial and 24-cm axial field of view was acquired, with a resolution of 1.5 × 1.5 × 2.0 mm^3^. The sequence consisted of two echoes (30 μs, 1.7 ms) with a flip angle of 10° and 125-kHz bandwidth. The CT images of all patients were classified by thresholding and used to compute maps of the posterior probability of each tissue class, given a pair of UTE echo values. The Jaccard distance was used to compare with CT the bone masks obtained when using this information to segment the UTE datasets.

**Results:**

The results show the desired bony structures as a cluster pattern in the space of dual-echo measurements. The clusters obtained for the tissue classes are strongly overlapped, indicating that the MR data will not, regardless of the chosen space partition, be able to completely differentiate the bony and soft structures.

The classification obtained by maximizing the posterior probability compared well to previously published methods, providing a more intuitive and robust choice of the final classification threshold. The distance between MR- and CT-based bone masks was 59% on average (0% being a perfect match), compared to 76% and 69% for two previously published methods.

**Conclusions:**

The study of tri-modality datasets shows that improved bone tissue classification can be achieved by estimating maps of the posterior probability of voxels belonging to a particular tissue class, given a measured pair of UTE echoes.

## Background

A necessary step to obtain quantitative positron emission tomography (PET) images is correcting for the signal attenuation introduced by the patient. In combined PET/computed tomography (CT) scanners, this can be achieved using the X-ray attenuation information provided by the CT. Combined PET/magnetic resonance (MR) scanners, on the other hand, rely on the segmentation of MR data into tissue classes (e.g., fat, soft tissue, lung) [1, 2]. Each class is then assigned a fixed attenuation value.

One yet unsolved problem with this approach is the difficulty of imaging and segmenting the bones. Conventional MR sequences are unable to detect the water bound to the organic matrix or the free water in the microscopic pores and canals of the osteons [3, 4]. This is due both to its intrinsically low proton density (≈20% of water content) and short signal lifetimes (T2 ≈ 390 μs at 3T).

Ultra-short echo time (UTE) MR sequences offer a potential solution to this problem. Several studies have already been published discussing the technical feasibility of UTE imaging for PET/MR attenuation correction [5–11]. One of the main issues of the proposed approaches is that they usually rely on theoretical models of the behavior of bone signal in MR. While generally correct, these simple models are insufficient to account for the variability found in practice, which leads to suboptimal segmentation. One notable exception is the approach developed by Larsson et al. [12], which uses combined CT and MR datasets to train their model. One drawback of this method is that it relies on a sophisticated combination of several MR acquisitions, only suitable for certain applications.

The present work evaluates a new segmentation approach of clinical UTE bone images, based on tissue probability maps obtained from concurrent CT data. For this purpose, a large set of oncology patients were analyzed, taking advantage of the availability of concurrent CT data acquired with a tri-modality (PET/CT + MR) setup. The main advantage of this approach, with respect to previously published methods, is that the rules for the segmentation are defined using measured data from the current gold standard for bone localization - CT datasets - rather than theoretical or empirical hypotheses. Furthermore, requiring only a single pair of UTE echoes for the segmentation, this approach is compatible with most clinical protocols. This also makes the relation between UTE and CT values easier to represent and interpret in terms of joint histograms and posterior probability maps.

Throughout this work, the focus was on head and neck imaging. The reason for this is twofold: Brain imaging is an important clinical application of PET, with a strong focus on quantitation and likely to gain further relevance in the near future for the detection of early-onset dementia. Furthermore, head imaging allows simplifying the problem, leaving aside issues like cardiorespiratory motion and unknown coil setup.

This study evaluates only the segmented bone masks obtained with the proposed method. Even if the ultimate goal of improving bone localization is enhancing MR-based attenuation correction, the final impact on PET image quality is not measured here, assuming that better bone masks will lead to better PET images in any case.

## Methods

### Scanner setup

The acquisitions were performed using a tri-modality setup consisting of a GE Discovery 750 w 3T MR system located in an adjacent room to a GE Discovery 690 ToF PET/CT (GE Healthcare, Waukesha, WI, USA). Patients were transported between the two systems using a dedicated transfer device (Innovation Design Center, Thalwil, Switzerland), enabling a consistent patient placement between the PET/CT and MR imaging systems [13]. An improved transfer shuttle that uses air pressure to facilitate the patient transfer was incorporated during the study, but should have no impact on the results.

### Patient population

Twenty patients referred for a clinical oncology PET/CT examination were acquired for this study. The average patient age was 62 ± 16 years [range 24 to 81], the average weight was 73 ± 14 kg [range 50 to 101], and the average body mass index was 25 ± 4 kg/m^2^ [range 17 to 34]. Two thirds of the patients were men and one third women [13/7]. The present study did not involve any extra radiation dose delivered to the patients, since the used CT was part of the clinical routine PET/CT examination. This study was approved by the institutional ethics committee, and written informed consent was obtained from all patients prior to the examination.

### Data acquisition

The PET/CT acquisition followed the standard protocol for a clinical oncology study. The average FDG dose was 266 MBq. Between six and eight bed positions were prescribed, covering head to mid-thighs. First, a helical CT scan (120 kV, 15 to 80 mA with automatic dose modulation, rotation time 0.5 s, helical thickness 3.75 mm, pitch 39.37 mm/rot, matrix size 512 × 512, 1.4 × 1.4 × 3.3 mm^3^) was acquired for attenuation correction of PET data. Then, each PET bed was acquired for a period of 2 min.

The voluntary MR examination was performed during the resting time after the injection of FDG, so the total time within the department experienced by the patient was not altered. This allowed approximately 30 min of MR scan time.

The Cones sequence was used for 3D-UTE acquisition [14]. This sequence employs *k*-space trajectories that sample data along twisting paths over evenly spaced cone surfaces, starting from the center of the *k*-space. This approach has a number of advantageous features, including enabling ultra-short echo times, efficient isotropic 3D sampling of data, and minimal diffusion sensitivity. The UTE acquisition lasted 320 s. This relatively long acquisition time was selected to provide information of the performance of UTE bone imaging, without compromises in resolution or *k*-space undersampling. A 22-cm transaxial and 24-cm axial field of view was acquired, with a resolution of 1.5 × 1.5 × 2.0 mm^3^. The sequence consisted of two echoes (TE1 30 μs, TE2 1.7 ms), with a flip angle of 10° and 125-kHz bandwidth.

The MR protocol included further sequences for the anatomical referencing of PET findings, not relevant for the present study.

### Algorithm design

The goal of this stage was to use the available CT information to achieve a better understanding of how different tissue classes are represented by dual-echo UTE imaging, to determine the optimal thresholds for segmentation.

**Figure 1:**
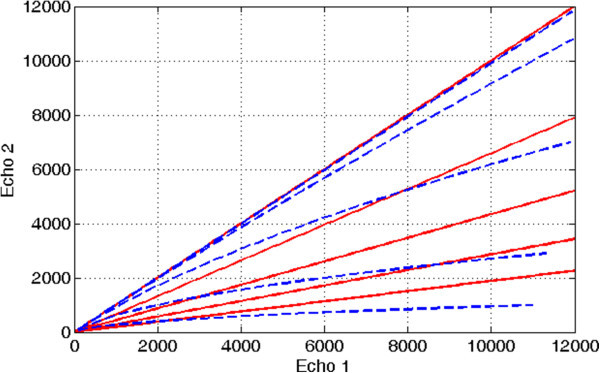
**Dual-echo UTE space**. Partitions of the dual-echo UTE space yielded by *R*_2_ thresholding (continuous lines) at 0, 250, 500, 750, and 1,000 s^-1^ and normalized difference thresholding (dashed lines) at 10^-2^, 10^-3^, 10^-4^, 10^-5^, and 10^-6^.

A usual way of detecting bone tissue in dual-echo UTE datasets, when no CT information is available, is to generate a *R*_2_ map:1

where *I*_TE1_ and *I*_TE2_ indicate the rooted-sum-of-square images with echo times TE1 and TE2, respectively. Notice that, in practice, this is a  map. Such a classifier (if thresholded) essentially yields straight cuts through the dual-echo space (Figure 1), intersecting the origin and decreasing their slope exponentially with the threshold value.

An alternative approach, also proposed in the literature, is to use the following normalization:2

This leads to a slightly curved cut through the dual-echo space (Figure 1), also intersecting the origin. As in the case of *R*_2_, the location of the cut varies exponentially with the threshold value. This makes the resulting classification quite sensitive to its parameters, having lost the easy physical interpretation that the *R*_2_ had.

In contrast to these methods, the approach proposed here exploits the availability of concurrently obtained CT and MR information from the tri-modality system. The segmentation of background, soft tissue, and bone tissue in CT is straightforward by intensity (Hounsfield unit (HU)) thresholding. By comparing this classification with the MR data, it is therefore possible to determine the most likely tissue class corresponding to each UTE echo pair.

As a first step in order to make this processing more repeatable, a patient-wise prescaling of both UTE echoes was performed to place the maximum of the soft tissue cluster on (4,000, 4,000). This maximum is easily identifiable in the joint histograms of the original echoes and can be reliably applied by an automated processing.

Additionally, an estimate of the acquisition noise was obtained by computing, for each patient and each UTE echo, the average and standard deviation of the image background. The background was defined, for each patient, as the intensity interval (0, *μ*_I_ + 3*σ*_I_), where *μ*_I_ and *σ*_I_ represent, respectively, the mean and standard deviation of all voxels where the intensity of the first UTE echo is smaller than 10^3^. This two-step method was chosen to center the measurement on the background peak of the histogram, rejecting partial volume effects and bone voxels.

An image registration package (Integrated Registration, AW workstation, GE Healthcare) was used to verify and adjust the registration between the patient head MR and CT images, for each clinical case. The included interactive rigid-body motion tool was used to ensure the proper alignment of the images, using salient bony structures as a reference (e.g., the lateral orbital pillar, superior part of the frontal sinus, internal occipital protuberance, and clivus). The CT datasets were then resampled to match the resolution of the MR images.

Once the CT and MR were properly aligned and resampled, it was possible to establish, for every UTE echo pair found in the data, the number of times (i.e., voxels) that it corresponded to each tissue type identified in the CT. By normalizing this information by the total number of occurrences of that echo pair, the posterior probability of each tissue class was generated (i.e., the probability of UTE echo pair (*x*, *y*) being obtained for air/bone/soft tissue). The total number of voxels per echo pair was clipped to a minimum value of 10, to eliminate outliers.

From each acquired patient, a map of the posterior tissue likelihood for each possible UTE echo combination was computed. The maps were then averaged over all patients, in order to determine the optimal classification thresholds to partition the space of dual UTE echoes into three classes: air, soft tissue, and bone tissue.

A certain number of *a priori* conditions were applied to these probability maps: Any voxels with Echo1 + Echo2 > 4,000 are never classified as air. Non-zero air probability in such voxels is assumed to be due to residual misregistration between our CT and UTE. Any voxels with Echo2 > Echo1 are never classified as bone. Non-zero bone probability in such voxels can be attributed to misregistration, patient motion, and partial volume effects. Any voxels with Echo1 > 8,000, Echo2 > 5,000, or Echo1-Echo2 > 4,000 are not classified as bone. Most of these cases have been found to be caused by either air/tissue interface effects or metal artifacts. Uncertain regions are labeled as soft tissue.

### Algorithm application

In clinical practice, the proposed algorithm requires only a dual-echo UTE acquisition to be performed. Each of the UTE images is then automatically rescaled to place the maximum soft tissue cluster on (4,000, 4,000). Then, the two echo values associated to each voxel are checked, and the voxel is assigned the tissue class with maximum posterior probability. In the current implementation, this is achieved using a lookup table, but faster implementations are certainly possible (e.g., modeling the bone and soft tissue clusters as ellipsoids).

The bone mask obtained by selecting all voxels with probability higher than 0.5 was compared to the masks obtained by selecting voxels with *R*_2_ higher than 0.35 and with *N* higher than 5 × 10^-4^. These values were selected empirically to achieve the best segmentation results over all patients. The Jaccard distance to the corresponding CT bone mask was used as a quality measure (with 0% distance corresponding to identical and perfectly aligned masks).

## Results

### Algorithm design

**Figure 2:**
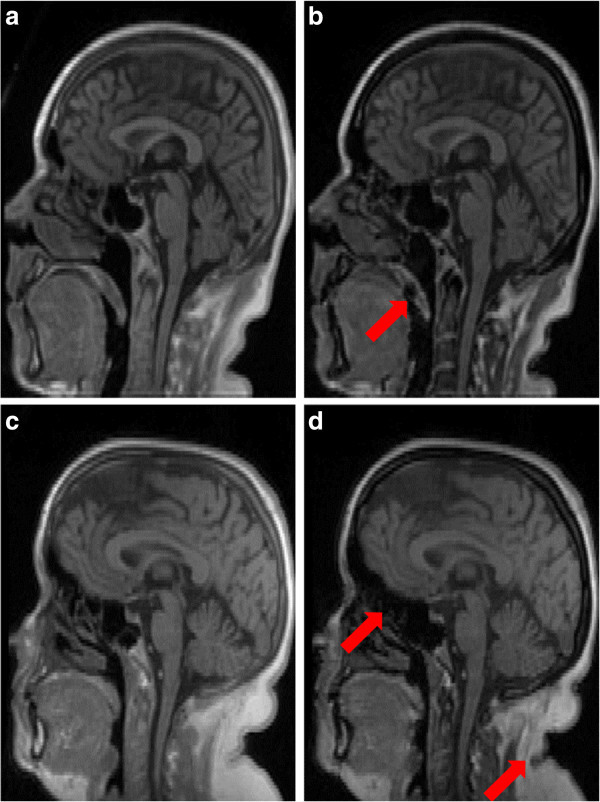
**Sagittal views, for two patients, of the measured UTE echoes. (a,** **c)** 30 μs and **(b,** **d)** 1.87 ms. The arrows indicate unwanted signal changes (e.g., swallowing motion and folds in the neck fat).

**Figure 3:**
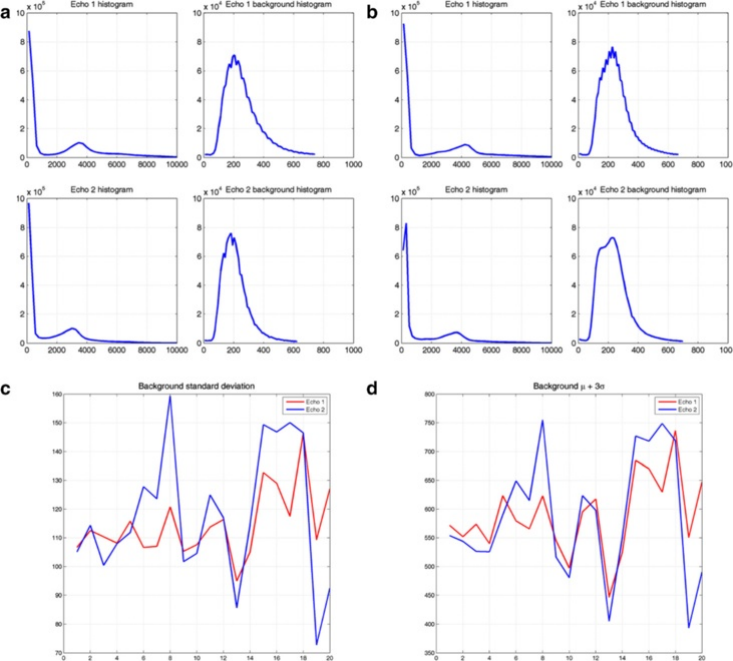
**Noise properties of the data. (a,** **b)** Background histograms of two patients, **(c)** background standard deviation for all patients, and **(d)** background average plus three times its standard deviation. The latter is provided as a reference to set the segmentation thresholds.

**Figure 4:**
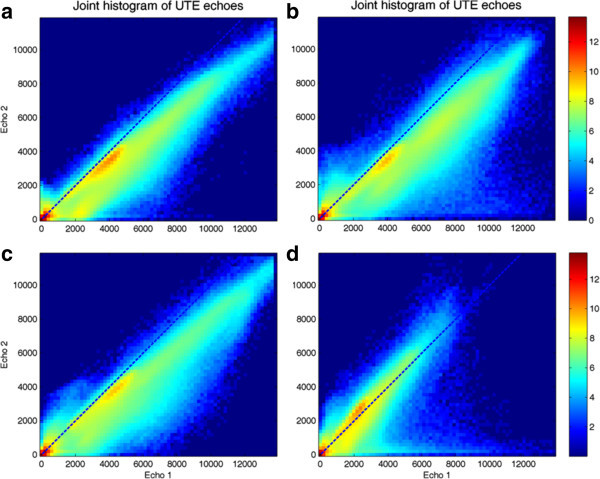
**Logarithmic plots of the joint histogram for four patients (a-d), before histogram peak correction**.

**Figure 5:**
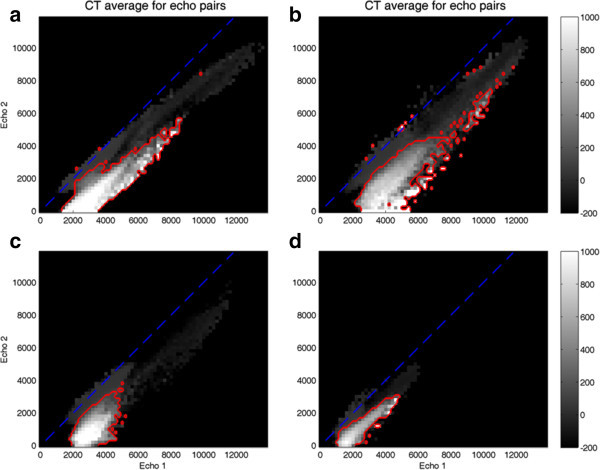
**Average CT value, for four patients (a-d), of all voxels sharing a specific UTE echo pair**. The red line delimits the region with an average CT value superior to +100 HU.

In order to illustrate the available data for MR-based bone segmentation, sagittal views of the dual UTE echoes have been included in Figure 2 for two typical cases. The arrows indicate unwanted signal changes that can lead to certain structures being incorrectly labeled as bone, such as swallowing motion and folds in the neck fat. The noise properties of the data can be appreciated in Figure 3, which illustrates the background histograms of two patients and the resulting background standard deviation for all patients. A plot of the background average plus three times its standard deviation is included as a reference to set the segmentation thresholds. Notice that, throughout this manuscript, MR image intensity values without units are used.Joint histogram analysis can be used to understand the behavior of the dual-echo pairs for different tissue types. Logarithmic plots of the joint histogram for four patients can be appreciated in Figure 4. Notice the distinct main soft tissue peak. Additional insight is provided by looking at the average CT value of all the voxels sharing a specific UTE echo pair (i.e., at each bin of the joint histogram), depicted in Figure 5. As a reference, air is at -1,000 HU, fat between -100 and -50 HU, water at 0 HU, brain matter between +20 and +45 HU, and (cortical) bone between +700 and +3,000 HU. The red line delimits the region with an average CT value superior to +100 HU (an upper threshold for soft tissue without contrast).

**Figure 6:**
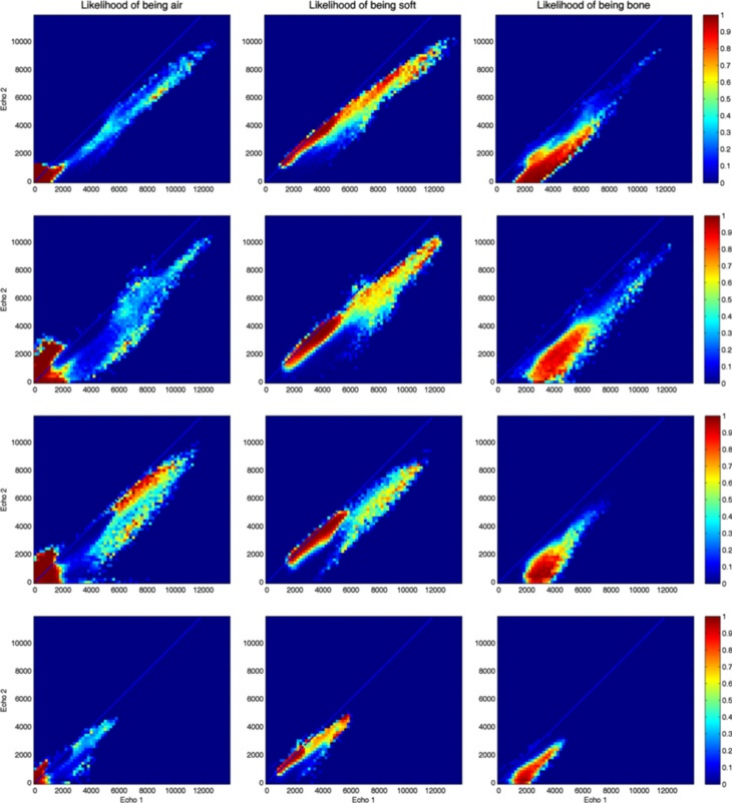
**Posterior probability maps of each tissue class (air, soft tissue, and bone) computed from four independent patient datasets**.

**Figure 7:**
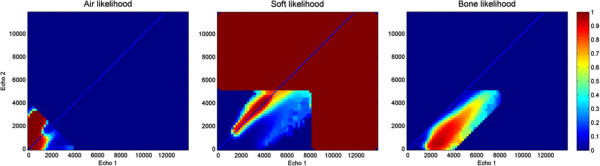
**Posterior probability maps of each tissue class (air, soft tissue, and bone) computed including all patient datasets**. *A priori* constraints have been imposed on the final maps.

By combining the information provided by CT and MR, it is possible to define the probability of any given UTE echo pair being generated by a certain tissue class. Figure 6 shows the posterior probability maps computed for each tissue class, for the same patients used in Figures 4 and 5. Notice the intrinsic uncertainty of the information provided by UTE, reflected by the overlap of soft and bone clusters. The posterior probability maps obtained by combining all the acquired patients can be appreciated in Figure 7. Notice how the *a priori* classification rules described in the previous section have been applied here. This information can then be used to define optimized segmentation thresholds.

### Algorithm application

**Figure 8:**
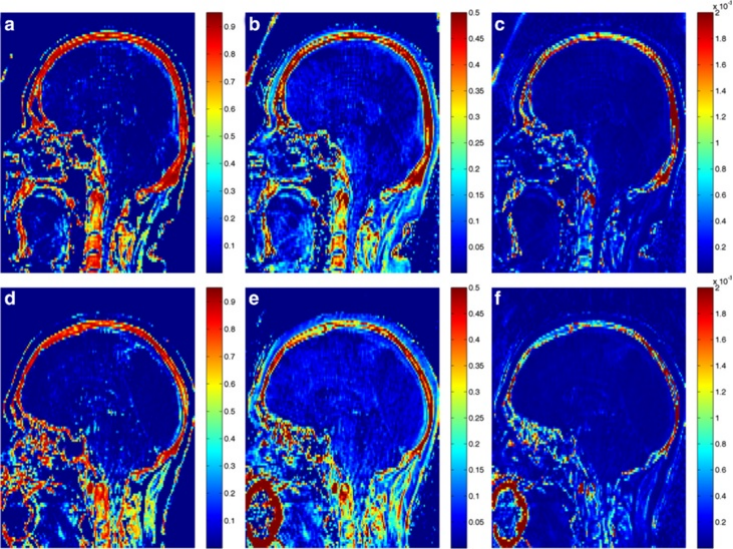
**Bone tissue maps**. The maps obtained, for two patients, with the **(a,** **d)** proposed approach compared with **(b,** **e)** Keereman's *R*_2_ method and **(c,** **f)** Catana's normalized difference method.

**Figure 9:**
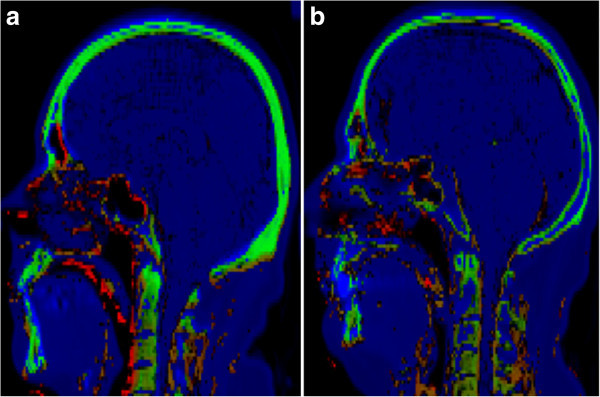
**Visual comparison of UTE cluster-based segmentation results with concurrently acquired CT data**. The color map reflects agreement between both modalities (green, true positive bone identification; red, false positive).

The results of the presented approach, compared with Keereman's *R*_2_ and Catana's normalized difference methods, can be appreciated in Figure 8. The threshold values for these methods were empirically set to achieve the best possible segmentation across all patients (without individual tuning). The average Jaccard distance using the proposed method was 59% (median 57%, standard deviation ±10%, range 49% to 88%), compared to 76% (76% ± 7%, 65% to 94%) using the *R*_2_ method and 69% (66% ± 9%, 56% to 90%) using the normalized difference method. A two-tailed *t* test assuming unequal variances was used to validate that these differences are statistically significant (*P* < 10^-3^ and *P* < 10^-2^, respectively).The output of our cluster-based segmentation method can also be visually compared, for two patients, to concurrently acquired CT data in Figure 9, using a two-dimensional color map that reflects the agreement between images (green representing true positive bone identification; red, false positive; blue, false negative; and black, true negative).

## Discussion

From the observation of the dual-echo UTE image pairs, the undesired behavior of the following elements, which are going to be the main obstacles for classification, can be noticed: low signal intensity of bone tissue, areas close to background, and body air (e.g., paranasal sinuses); high difference values of non-osseous tissue (e.g., tendons); high difference values due to local field inhomogeneities, ringing, partial volume, and patient motion; non-uniform bone values due to partial volume and shading; and presence of local coil components in the background.Overall, the proposed method generated satisfactory bone maps that are coherent with the concurrently acquired CT datasets. Notice that some of the discrepancies, such as those found in the vertebrae and skull cavities in Figure 9, are due to residual misregistration rather than to bone classification errors. The issues of dental artifacts and the presence of coil structures are significantly reduced, whereas the misclassification of certain tendons and cartilage remains strongly dependent on the choice of threshold value. The use of two-step thresholding techniques to address this problem will be tested in a follow-up study. Similarly, spurious voxels in tissue-air interfaces are present in the results but readily addressed with simple morphology-based post-processing.

This cluster-based method for the localization of bone tissue has been tested on head/neck imaging but should be straightforward to apply to other non-moving areas. The current study was performed on a GE Healthcare 3T scanner, but the method is expected to work on any scanner equipped with a 3D ultra-short echo imaging sequence, both at 3T and 1.5T, once the posterior probability maps are recomputed [15].

Concerning the differences between the proposed posterior probability-based method and previously published methods - such as Keereman's *R*_2_ [5] and Catana's normalized difference maps [7] - they are necessarily subtle: with suitable threshold values (consider Figure 1), all methods are capable of capturing the main peak of the bone cluster, as depicted in Figure 5. There are, however, advantages to using well-defined prior information of the distribution of each tissue cluster, based on actual CT measurements, rather than pure theoretical or empirical threshold functions. One such advantage would be the ease and robustness of selecting the final classification thresholds (again, we refer the reader to Figure 1). Indeed, the probabilistic formulation provides a well-understood context for the further improvement of the algorithm, easily scalable to incorporate new tissue classes and additional constraints. Additionally, it offers possibility of further exploiting the information provided by the CT training set to provide a local estimation of the attenuation coefficient of bone.

Even if these advantages with respect to previously published methods seem technically subtle, they might translate into concrete advantages in clinical PET/magnetic resonance imaging (MRI). The additional information deriving from dual-echo UTE could be of significant value for applications where, e.g., accurate quantitation in the presence of large bony structures is required, such as brain or prostate radiotherapy. Especially, the latter one is of larger clinical interest since prostate cancer represents one of the most common cancers in western industrialized countries and radiotherapy is one of most chosen therapeutic options, especially in cases of recurrence. Since PET/MRI holds the promise for integrated and potentially improved diagnostic accuracy in prostate cancer by combining the high soft tissue contrast of MRI with the characteristics of new tracers (e.g., PMSA), it is obvious and mandatory to have reliable attenuation correction and signal quantification for those procedures. Also for therapeutic trials where robust SUV quantification is needed for longitudinal follow-up, such an improved approach will have impact on the reliability of the long-term results. This would make PET/MRI trials especially more attractive for pharmaceutical trials, where quantification of PET (whether it is PET/CT or potentially PET/MRI) suffers from greater variation, e.g., compared to computed tomography and Hounsfield units.

An extension of the proposed method is indeed possible by including additional MR sequences, along the lines of the algorithm presented by Johansson et al. [9]. This could certainly improve the segmentation results, e.g., reducing the number of false positive elements in the bone mask, limiting the extent of artifacts caused by metallic implants, or enabling the differentiation of fatty tissue. Such an extension would, however, limit the usability of the method to single-station protocols with sufficient time for MR acquisition. Furthermore, the posterior probability approach has the advantage of yielding more intuitive results than more advanced (and less transparent) machine learning methods. This improved understanding of the classification problem enables the development of further enhancements to the segmentation algorithms, as well as the extension to new applications without the need for a new full training dataset.

Last but not least, since all PET/MRI systems are in their early clinical research stages, not much experience is currently available with off-norm patients, such as those operated and/or with significantly altered bony structure (e.g., after brain surgery). In such a patient population, robust quantification and safe classification of bone compared to surrounding anatomical structures is critical for accurate diagnosis.

In any case, the method and the optimal settings for it still need to be tested prospectively. The next step must therefore be its validation within a complete PET attenuation correction and reconstruction pipeline, in order to determine whether the differences reported here with respect to previously published approaches do indeed lead to a significant improvement in the resulting emission images.

## Conclusions

In this study, tri-modality data have been used to develop and validate an improved approach for the segmentation of bone tissue from dual-echo ultra-short echo MR data. The distribution of the different tissue classes in the dual-echo space could be obtained thanks to the availability co-registered CT data. This information enables the implementation of accurate bone segmentation for the purpose of PET/MR attenuation correction, offering a more intuitive formulation than previously published approaches and more robust definition of the segmentation threshold. Future work will focus on further exploiting tissue distribution maps with the goal of replacing the current binary bone masks with actual estimations of bone tissue density.
